# Emergency Department–Initiated Tobacco Control: Update of a Systematic Review and Meta-Analysis of Randomized Controlled Trials

**DOI:** 10.5888/pcd14.160434

**Published:** 2017-10-05

**Authors:** Christina Lemhoefer, Gwen Lisa Rabe, Jürgen Wellmann, Steven L. Bernstein, Ka Wai Cheung, William J. McCarthy, Susanne Vahr Lauridsen, Claudia Spies, Bruno Neuner

**Affiliations:** 1 Charité – Universitätsmedizin Berlin, Department of Anesthesiology and Intensive Care Medicine, Berlin, Germany; 2 Krankenhaus der Augustinerinnen, Department of Internal Medicine, Cologne, Germany; 3 Institute of Epidemiology and Social Medicine, University of Münster, Münster, Germany; 4 Department of Emergency Medicine, Yale University School of Medicine; Yale Cancer Center; Department of Health Policy, Yale School of Public Health, New Haven, Connecticut; 5 Department of Emergency Medicine, Faculty of Medicine, University of British Columbia, Vancouver, British Columbia; 6 University of California Los Angeles, Center for Cancer Prevention and Control Research, Fielding School of Public Health and Jonsson Comprehensive Cancer Center, Los Angeles, California; 7 University Hospital of Copenhagen, Rigshospitalet, Department of Urology, Copenhagen, Denmark

## Abstract

**Introduction:**

A 2012 systematic review and meta-analysis of randomized controlled trials on emergency department–initiated tobacco control (ETC) showed only short-term efficacy. The aim of this study was to update data through May 2015.

**Methods:**

After registering the study protocol on the international prospective register of systematic reviews (PROSPERO) in May 2015, we searched 7 databases and the gray literature. Our outcome of interest was the point prevalence of tobacco-use abstinence at 1-month, 3-month, 6-month, or 12-month follow-up. We calculated the relative risk (RR) of tobacco-use abstinence after ETC at each follow-up time separately for each study and then pooled Mantel–Haenszel RRs by follow-up time. These results were pooled with results of the 7 studies included in the previous review. We calculated the effect of ETC on the combined point prevalence of tobacco-use abstinence across all follow-up times by using generalized linear mixed models.

**Results:**

We retrieved 4 additional studies, one published as an abstract, comprising 1,392 participants overall. The 1-month follow-up point prevalence of tobacco-use abstinence after ETC resulted in an RR of 1.49 (95% confidence interval [CI], 1.08–2.05) across 3 studies; 3-month follow-up, an RR of 1.38 (95% CI, 1.12–1.71) across 9 studies; 6-month follow-up, an RR of 1.09 (95% CI, 0.84–1.41) across 6 studies; and 12-month follow-up, an RR of 1.26 (95% CI, 1.00–1.59) across 3 studies. The effect on the combined point prevalence of abstinence was an RR of 1.40 (95% CI, 1.06–1.86) (*P* = .02).

**Conclusion:**

ETC is effective in promoting continual tobacco-use abstinence up to 12 months after intervention. ETC may be a critically important public health strategy for engaging hard-to-reach smokers in tobacco-use cessation.

## Introduction

In 1998, a task force of the Society for Academic Emergency Medicine published recommendations for screening and intervention activities in emergency departments (EDs), including smoking cessation counseling (1,2). In 2006, a panel convened by the American College of Emergency Physicians called on emergency care providers to routinely screen ED patients for their smoking status and to initiate smoking cessation counseling or referral to outpatient treatment or both (3). These ED–initiated tobacco control (ETC) services are meant to reduce the burden of tobacco-related diseases by using the teachable moment of the ED visit to motivate smokers to quit (3,4).

Although several compelling arguments exist for such services, including that the prevalence of smoking is high among ED patients (5–7), typical ED patients are hard to reach, and EDs have high levels of credibility on the topic of preventive and health promoting services (8), the benefit of ETC is unclear.

A 2008 systematic review of smoking cessation interventions in the ED (9) identified 7 studies; only one reported a significant intervention benefit. Likewise, a systematic review and meta-analysis that was published in 2012 and that covered publications through October 2010 found a point-prevalence abstinence benefit of ETC over usual care only at 1 month after ETC. That study also found a nonsignificant effect (*P* = .08 across 7 studies) for a cumulative point-prevalence abstinence benefit of ETC over all follow-up points (1 month, 3 months, 6 months, and 12 months after ETC) (10). In 2014, a systematic review identified 13 randomized controlled trials (RCTs); 11 trials reported no significant differences between study groups (11). Although persuasive evidence exists on the efficacy of tobacco control interventions in other medical settings (12,13), the efficacy of ETC on cessation rates for periods exceeding 1 month has yet to be demonstrated. The objective of our study was to provide an update of the systematic review and meta-analysis of RCTs published in 2012 (10).

## Methods

### Data sources

This systematic review and meta-analysis updates a previous review, which comprised 1,986 participants overall and included articles published through October 4, 2010 (10). Inclusion criteria, quality assessment, and analysis methods were identical to those used in the previous review (10); all methods comply with the Preferred Reporting Items for Systematic Reviews and Meta-Analyses (PRISMA) statement (14). Details of our protocol were registered on the international prospective register of systematic reviews (PROSPERO) in May 2015 and can be accessed at www.crd.york.ac.uk/PROSPERO/display_record.asp?ID=CRD42015020581.

The systematic search was conducted by 1 reviewer (C.L.) in 7 electronic databases: MEDLINE, The Cochrane Library, EMBASE, PsycINFO, Scopus, LILACS (15), and the citation indexes of the ISI Web of Knowledge. We screened the International Clinical Trial Registry Platform for unpublished studies, the Conference Proceedings Citation Index, which contains information on gray literature and unpublished studies, and the references of the included studies to identify additional potentially relevant studies.

The following search terms were used: (Smok* AND Emergency OR Tobacco AND Emergency OR Nicotine AND Emergency OR Cigarette* AND Emergency) AND (Control OR Intervention* OR Counseling OR Counselling OR Assistance OR Treat* OR Prevention OR Promotion* OR Referral* OR Cessation) AND (randomized OR randomly OR control* OR trial* OR controlled study OR investigation OR prospective OR longitudinal OR pilot).

### Study selection

We searched for studies published between October 4, 2010, and May 15, 2015. Studies had to be accessible and published at least as an abstract in English or Spanish. Inclusion criteria were RCTs with ED patients of any age who were current smokers and who were offered a tobacco control intervention. We defined smoking cessation interventions according to the American College of Emergency Physicians statement (3) as motivational interviewing or counseling on site in combination with referral to outpatient treatments or to telephone quitlines. The treatment in the control group could be usual care (receipt of brochures, self-help material, information leaflets on state smokers’ quitlines, or any less intensive program such as brief advice only or no material or advice at all). The outcome — usually evaluated as self-reported 7 days of tobacco-use abstinence (point-prevalence tobacco-use abstinence [16]) — had to be measured at least once during follow-up. We excluded studies of patients from outpatient settings, studies of relatives or visitors of ED patients, and studies of hospitalized patients.

Relevant text was imported into Reference Manager Version 12.0 (Alfasoft GmbH). After duplicate removal, 2 reviewers (C.L. and G.L.R.) independently screened titles and abstracts of all remaining search results for relevance. In a second step, the full texts of potentially relevant studies were assessed for eligibility. Any disagreement between reviewers was resolved in discussion with a third reviewer (B.N.).

### Data extraction

Two investigators (C.L. and B.N.) extracted relevant information on study design and outcomes independently. The following data were extracted: 1) setting (type of ED, location, annual patient census), 2) participants (total number in study, number in each study arm, age and sex distribution), 3) smoking-related variables (smoking definition, screening instruments, biologic validation, and use of other biological markers [data not tabulated for this article]), 4) type of smoking cessation intervention and type of treatment in the control group, 5) follow-up (time in months, number of follow-up contacts, absolute number and percentage of participants lost to follow-up, number of abstinent smokers [or other outcomes] at each follow-up).

Risk of bias was assessed independently by two reviewers (C.L. and B.N.) by employing the Quality Assessment Tool for Quantitative Studies (17). The tool includes ratings on the following 6 components: selection bias, study design, confounders, blinding, data collection methods, and withdrawals and dropouts. Combined component ratings resulted in a global rating of either “strong,” “moderate,” or “weak.” We assessed potential publication bias by constructing a funnel plot of the RRs that compared the benefit of ETC with the control condition; the plot used data from the longest follow-up point in each study. We used the Peters test to evaluate the symmetry of the funnel plot (18).

### Data synthesis

Although the number of follow-up contacts varied among studies, all studies reported abstinence rates at 1-month, 3-month, 6-month, or 12-month follow-up. Study participants not reached at follow-up were assumed to be current smokers. We calculated the proportion of tobacco abstainers in both study arms for each study for each follow-up time. The first analysis across studies consisted of pooling the relative risk (RR) of abstinence (which represents the relative benefit of the smoking cessation counseling (ie, the ratios of the proportions of abstainers in the treatment group to the proportions of abstainers in the control group) across studies by using Mantel–Haenszel RRs. However, our main meta-analysis used the individual study results for each follow-up. To account for heterogeneity among studies and repeated measurements within studies, we used generalized linear mixed models (19,20) with 7-day point-prevalence tobacco-use abstinence at all follow-up times as the outcome. We used random intercepts to model variability in smoking status across studies as a function of ETC treatment versus no ETC treatment in interaction with time of assessment. A log link and a binomial error distribution were used to estimate the log odds of RRs; exponentiation was then used to report the RRs and their 95% confidence intervals (CIs). The variance–covariance matrix used reflected the fact that outcomes were highly correlated across follow-up times within each study, because the repeated assessments of smoking behavior involved the same participants. The final generalized linear mixed model was set up with 4 fixed effects (the intercept, the treatment effect, the effect of time, and their interaction). Additionally, we ran sensitivity analyses in subgroups, for example, involving studies that featured on-site motivational interviewing in combination with booster telephone calls. *P* < .05 was defined as significant.

## Results

**Figure 1:**
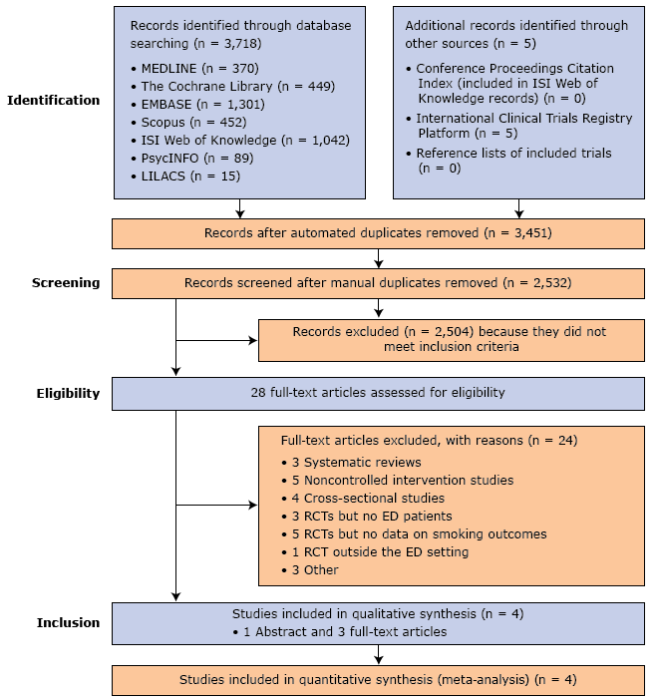
Flowchart showing the literature search in 7 electronic databases and the sequential study selection process. Abbreviations: ED, emergency department; EMBASE, Excerpta Medica database; LILACS, Literatura Latino-Americana e do Caribe em Ciências da Saúde (Literature in the Health Sciences in Latin America and the Caribbean); MEDLINE, MEDical Literature Analysis and Retrieval System Online of the United States National Library of Medicine; PsycINFO, literature database of the American Psychological Association; RCT, randomized controlled trial.

The literature search initially identified 3,723 studies. Of these, 2,532 remained after duplicate removal. A further 2,504 studies did not meet the inclusion criteria. Of the remaining 28 publications, 24 were excluded for other reasons (Figure 1), including 1 qualitative study and 2 quasi-RCTs (21–23).

Four studies consisting of 1 abstract and 3 full-text articles and comprising 1,392 participants were included in the systematic review and meta-analysis (24–27). Follow-up time ranged from 1 month to 12 months.

### Eligible studies

All 4 studies featured on-site motivational interviewing or cessation advice; 3 studies also featured booster telephone calls (Table 1). Anders et al (24) tested the feasibility and effectiveness of giving brief advice on site in combination with booster telephone calls. An external telephone counseling service delivered these calls within 2 weeks of the brief advice. Additionally, up to 6 cognitive–behavioral therapy sessions, either telephone based or in person, and nicotine replacement therapy (NRT) were provided gratis. Control group participants received 1 session of personalized cessation advice and printed self-help materials. Follow-up occurred 3 months later. Both studies by Bernstein et al (25,26) tested a combination of on-site motivational interviewing and booster telephone calls within 3 days. Additionally, study participants were offered printed materials and NRT gratis for up to 6 weeks. The comparison group received a brochure and quitline information only. Biochemically confirmed tobacco-use abstinence rate at 3 months was the primary end point. The second study by Bernstein et al, in 2015, also evaluated abstinence rates at 12 months (26). Cheung et al (27) explored a brief on-site intervention followed by referral to a provincial telephone quitline; abstinence rates were evaluated at 1-month, 3-month, 6-month, and 12-month follow-up.

**Table 1:** Characteristics of the 4 Studies Retrieved by a Systematic Review of Studies Describing Emergency Department–Initiated Tobacco Control^a^

Study Characteristic	Anders et al, United States, 2011^b^	Bernstein et al, United States, 2011^c^	Cheung et al, Canada, 2013^d^	Bernstein et al, United States, 2015^e^
**No. of participants randomized (intervention/ control) [target group]**	221 (111/110) [Adults]	338 (170/168) [Adults]	53 (27/26) [Adults]	778 (388/390) [Adults]
**Setting (estimated no. of patients annually)**	Urban emergency department (48,000)	Urban emergency department (90,000)	Urban emergency department (85,000)	Urban emergency department (90,000)
**Smoking definition**	Answer of yes to the question “Do you smoke tobacco?” and smokes ≥1 cigarette per day	Smoked >100 cigarettes in lifetime and current daily or occasional smokers with a mean consumption of ≥10 cigarettes per day	Tobacco use within the previous 30 days	Smoked >100 cigarettes in lifetime and current or occasional smokers with a mean consumption of >5 cigarettes per day
**Treatment in the intervention group**				
Advice and/or motivational interviewing on site	Yes	Yes	Yes	Yes
Booster telephone calls	Yes	Yes	No	Yes
Free nicotine replacement therapy	Yes	Yes	No	Yes
Brochure	No	Yes	No	Yes
**Treatment in the control group**	1) Personalized advice to quit smoking given by an advanced practice nurse and 2) self-help material plus brochure with contact information for cessation a program	Brochure with general information about smoking cessation and contact information for smoking cessation programs	Usual practice only	1) Brochure with general information about smoking cessation and 2) telephone number of the state smokers’ quitline
**Definition of tobacco-use abstinence**	Self-reported 7 days of abstinence	Self-reported 7 days of abstinence, verified by exhaled carbon monoxide and salivary cotinine	30-day point-prevalence abstinence	Self-reported 7 days of abstinence, verified by exhaled carbon monoxide

a This systematic review and meta-analysis updates a previous review (10) and includes publications published between October 4, 2010, and May 15, 2015.

b Anders et al (24).

c Bernstein et al (25).

d Cheung et al (27).

e Bernstein et al (26).

The proportion of abstinent smokers ranged from 4.9% to 34.6% in the studies analyzed (Table 2). In 2 studies, smokers in the intervention group had higher abstinence rates than did smokers in the control group (25,26). Anders et al (24) demonstrated that all participants in the intervention group consented to a faxed referral, of whom 13.5% attended treatment sessions, whereas 2.7% of the control group attended treatment sessions. Cheung et al (27) found that 16 of 27 (59.3%) participants in the intervention group accepted a referral to the quitline; 6 participants were reached, but only 5 enrolled in the program and only 2 completed the program.

**Table 2:** Number and Proportion of Abstinent Smokers at Follow-Up in 4 Studies Retrieved By a Systematic Review of Studies Describing Emergency Department–Initiated Tobacco Control^a^

Study (Year of Publication)	Type of Group	No. of Randomized Participants	No. (%) Abstinent Smokers at Follow-Up^b^			
			1 Month	3 Months	6 Months	12 Months
Anders et al (2011)^c^	Intervention	111	—	5 (4.5)	—	—
	Control	110	—	8 (7.3)	—	—
Bernstein et al (2011)^d^	Intervention	170	—	25 (14.7)	—	—
	Control	168	—	22 (13.2)	—	—
Cheung et al (2013)^e^	Intervention	27	7 (25.9)	8 (29.6)	6 (22.2)	4 (14.8)
	Control	26	4 (15.4)	4 (15.4)	9 (34.6)	7 (26.9)
Bernstein et al (2015)^f^	Intervention	388	—	47 (12.1)	—	62 (16.0)
	Control	390	—	19 (4.9)	—	45 (11.5)

a This systematic review and meta-analysis updates a previous review (10) and includes publications published between October 4, 2010, and May 15, 2015.

b Dashes indicate that study did not collect follow-up data at that point.

c Anders et al (24).

d Bernstein et al (25).

e Cheung et al (27).

f Bernstein et al (26).

### Risk of bias assessment

When we used the Quality Assessment Tool for Quantitative Studies to assess the risk of bias associated with the 3 full-text studies, 2 studies received a moderate rating (24,26), and 1 study received a weak rating (25). The study by Cheung et al (27) could not be assessed because of insufficient information.

### Update of the systematic review and meta-analysis

When we added the evidence from the newly retrieved studies to the evidence from the previous meta-analysis (10), we found that pooled results at 1 month after ETC (*P* = .01) and at 3 months after ETC (*P* = .003) were significant (Table 3). At 12 month follow-up, the pooled results were not significant (*P* = .05). Pooling all available evidence from all follow-up assessments across 11 studies, the cumulative point-prevalence abstinence of ETC compared with the control condition yielded an RR of 1.40 (95% CI, 1.06–1.86) (*P* = .02). Excluding the study by Cheung et al, which was reported as an abstract only, the cumulative point-prevalence abstinence of ETC compared with the control condition yielded an RR of 1.36 (95% CI, 1.00–1.85) (*P* = .047). Pooling the 8 studies that evaluated on-site motivational interviewing compared with booster telephone calls (24–26,30–34), yielded an RR of 1.39 (95% CI, 1.00–1.92) (*P* = .048). Further sensitivity analyses showed that after pooling only the 4 newly retrieved studies, the cumulative point-prevalence abstinence of ETC compared with the control condition yielded an RR of 1.57 (95% CI, 0.59–4.17) (*P* = .24). Pooling the 4 studies with biochemically confirmed smoking outcomes (25,26,32,34), the cumulative point-prevalence abstinence of ETC compared with the control condition yielded an RR of 1.34 (95% CI, 0.91–1.97) (*P* = .10).

**Table 3:** Benefit of Emergency Department-Initiated Tobacco Control Compared With Control Condition on Tobacco-Use Results of Individual Studies (N = 11) and Meta-Analyses, by Follow-Up Time^a^

Year of Publication, Study	Mantel–Haenszel Relative Risk (95% Confidence Interval)			
	1 month	3 months	6 months	12 months
2000, Antonacci and Eyck (28)	—	—	0.33 (0.01–7.74)^b^	—
2000, Richman et al (29)	—	1.14 (0.36–3.57)	—	—
2007, Horn et al (30)	—	—	0.83 (0.05–12.77)	—
2007, Schiebel and Ebbert (31)	—	2.00 (0.20–20.33)	9.00 (0.52–156.91)^b^	—
2008, Bock et al (32)	1.64 (1.04–2.56)	1.35 (0.86–2.12)	1.04 (0.64–1.68)	—
2008, Boudreaux et al (33)	—	1.86 (0.25–13.91)	—	—
2009, Neuner et al (34)	1.30 (0.79−2.15)	1.13 (0.75–1.69)	1.14 (0.81–1.61)	1.25 (0.91–1.72)
2011, Anders et al (24)	—	0.62 (0.21–1.83)	—	—
2011, Bernstein et al (25)	—	1.12 (0.66–1.91)	—	—
2013, Cheung et al (27)	1.69 (0.56–5.08)	1.93 (0.66–5.63)	0.64 (0.27–1.55)	0.55 (0.18–1.66)
2015, Bernstein et al (26)	—	2.49 (1.49–4.16)	—	1.38 (0.97–1.98)
Meta analyses	1.49 (1.08–2.05) [*P* = .01]	1.38 (1.12–1.71) [*P* = .003]	1.09 (0.84−1.41) [*P* = .54]	1.26 (1.00–1.59) [*P* = .05]

a This systematic review and meta-analysis updates a previous review (10) and includes publications published between October 4, 2010, and May 15, 2015.

b 0.5 added to all cells of the 2 × 2 table in calculating the relative risks to avoid degeneracy caused by sampling zero counts.

**Figure 2:**
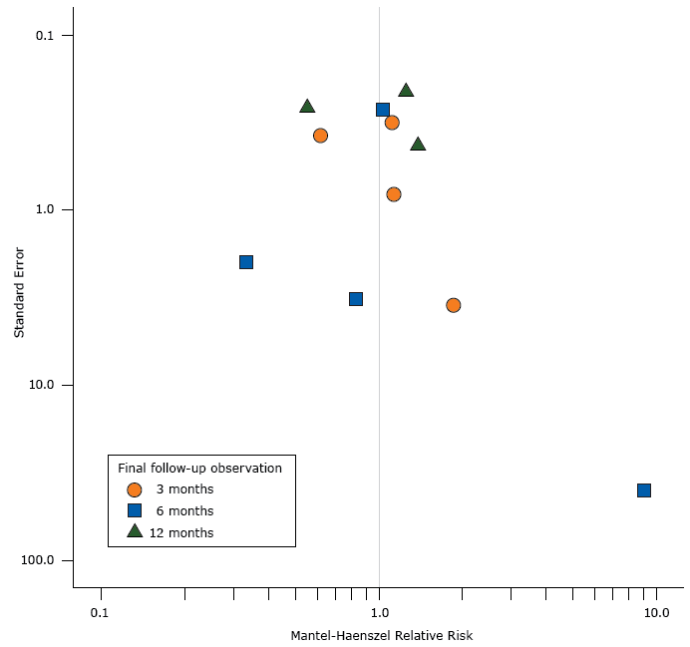
Funnel plot showing the effect estimates (Mantel-Haenszel relative risks/benefits of emergency department–initiated tobacco control) on the *x*-axis and the standard errors of the effect estimates on the *y*-axis. The funnel plot used data from the final follow-up observation in 11 studies. Both axes are log-10 scales. **Table:** 

The funnel plot, based on 11 observations, was not significantly asymmetric (*t*_11_ = −0.57, *P* = .58) (Figure 2). Overall, 7 studies had positive results (RR > 1), some of which had large standard deviations; however, the 4 studies with negative results (RR < 1) show both large and small standard deviations.

Study and Year	Final Observation	Mantel-Haenszel Relative Risk	Standard Error
Antonacci and Eyck, 2000	6 month	0.33	1.97
Richman et al, 2000	3 month	1.14	0.82
Horn et al, 2007	6 month	0.83	3.24
Schiebel and Ebbert, 2007	6 month	9.00	39.90
Bock et al, 2008	6 month	1.04	0.27
Boudreaux et al, 2008	3 month	1.86	3.48
Neuner et al, 2009	12 month	1.25	0.21
Anders et al, 2011	3 month	0.62	0.41
Bernstein et al, 2011	3 month	1.12	0.32
Cheung et al, 2013	12 month	0.55	0.38
Bernstein et al, 2015	12 month	1.38	0.26

## Discussion

This update of a systematic review and meta-analysis of RCTs evaluating the efficacy of ETC indicated an overall benefit of ETC over the control condition on repeated 7-day point-prevalence abstinence at 1-month, 3-month, 6-month, and 12-month follow-up. At 1-month and 3-month follow-up, the pooled 7-day point-prevalence abstinence was higher in the ETC group than in the control group and showed a tendency to be higher at 12-month follow-up. Eight (24–26,30–34) of 11 studies evaluated interventions that combined on-site motivational interviewing with booster telephone calls. Pooling these studies, ETC showed higher cumulatively assessed point-prevalence abstinence than did the control condition.

The quality standard of our systematic review was similar to that of Rabe et al (10): our literature search encompassed the 7 most relevant major electronic databases and unpublished studies. We documented the methodological quality and the risk of bias for all 11 studies included in our review. The statistical approach replicated that used in our earlier systematic review. We conservatively assumed that all participants lost to follow-up were smoking at follow-up. We found some heterogeneity in the intervention strategies used in the studies. However, all but 1 of the newly retrieved studies and 8 of the 11 studies in the review investigated the impact of the combination of motivational interviewing and advice to quit on site or through booster telephone calls delivered promptly after the ED treatment. In 3 of 4 of the newly retrieved studies, study participants received NRT gratis. Heterogeneity mainly occurred in the provision of self-help materials and brochures and referrals to telephone quitlines. Our statistical approach accounted for this heterogeneity in the calculation of the overall effect of ETC. Our approach allowed for between-study variance and within-study variance over time and may therefore satisfactorily reflect the true variability of intervention conditions in clinical practice. The funnel plot showed that 4 (of 11) studies with negative results were indeed published, although 7 (of 11) studies had positive results. The overall shape of the plot was fairly symmetric (with predictably greater heterogeneity of effect sizes among the smaller studies, as reflected in the expected inverted funnel shape [35]). This lack of funnel plot asymmetry was confirmed statistically. Despite the foregoing, publication bias cannot be ruled out as the explanation for these data, but it seems unlikely. Pooling results of the newly retrieved studies showed an effect size larger than that found by the previous meta-analysis (10). Thus, the more impressively significant results of the current update are attributable to both the increased cumulative sample size made possible by the addition of the newly retrieved studies and to the larger effect sizes reported by these recent studies. Abstinence rates at 6 months and 12 months in the newly retrieved studies with larger sample sizes compared favorably with the results of the previous meta-analysis (10). One possible reason for this improvement is the more consistent delivery of NRT in the more recent trials. Not all studies validated smoking outcomes biologically. The pooled result on the cumulative point-prevalence abstinence of ETC in studies with validated tobacco abstinence was slightly weaker (RR = 1.34) than the pooled result of all available studies (RR = 1.40). Although several studies that examined the validity of self-reported smoking data concluded that self-reported smoking history was accurate (36,37), our finding may indicate some social desirability bias in the nonvalidated studies and thus may slightly overestimate the true effect of ETC.

From a public health point-of-view, a measure of continuous tobacco-use abstinence would provide more unambiguous evidence of long-term ETC efficacy than the measure of point-prevalence abstinence used here (13,15). However, insisting on strictly continuous abstinence may unfairly classify too many successes as relapses (15,38). It is common research practice to assess 7-day point-prevalence rates supported by a negative biochemical test (15), because these data are regarded as valid, replicable outcome measures and less likely to be biased by faulty recall or social desirability.

This meta-analysis showed a nonmonotonic attenuation of the ETC effect over 12 months. We believe that the apparent drop in effect size at 6 months was artifactual and does not represent the 12-month trend. Of 6 studies pooled at 6 months, 3 studies (27,28,30) showed negative results. Two of these studies (28,30) reported no 12-month results. The third study (27) found an advantage of ETC over the control condition at 1 month and 3 months but not thereafter. Thus, the attenuation at 6 months was driven by 2 studies with 6-months–only results and by 1 small study, which attenuated the pooled 12-month results negligibly. However, a drop-off of intervention effect over time is characteristic of most interventions designed to reduce psychoactive drug use (39) or tobacco use (38). Without changing the conditions that gave rise to smoking in the first place, relapse appears to be the rule. Even if tobacco abstinence is time-limited, there is nonetheless a significant benefit to the smoker’s lung health in having enjoyed a respite from smoking’s daily assault on normal physiological functioning (40). Moreover, previous experience with quitting seems to predispose to further attempts to quit (38).

Our review and meta-analysis strengthen and extend the evidence for the beneficial impact of smoking cessation interventions in EDs. The addition of the most recent studies has enriched the portfolio of novel approaches, especially booster sessions after the initial contact in the ED. Anders et al (24) used cessation services that offered 2 telephone calls within 2 weeks after ED treatment. A similar approach was chosen by Cheung et al (27) and Bernstein et al (25,26), who referred patients to a telephone quitline service. Such services have shown their feasibility and effectiveness in tobacco control in other settings, such as primary care practices (41). The use of telephone quitline services might reduce the workload of EDs that do not have such follow-up services available. Anders et al (24) and Bernstein et al (25,26) provided NRT gratis to their patients. Participants with higher levels of nicotine dependence were less likely to benefit from exposure to tobacco counseling in EDs or other settings (34,42–44). Additionally, providing NRT during cessation counseling (alone or in combination with other pharmacologic strategies) increased the number of quit attempts and abstinence rates in certain patient groups (45,46). Because we had no individual data and thus no information on the actual use of NRT in the populations studied in this systematic review and meta-analysis, we could not identify the attributable benefit of NRT provided gratis on near-term or long-term cessation outcomes.

Our meta-analysis suggests that cessation counseling initiated in the ED promotes repeated tobacco abstinence. Because of their high levels of reach and the high percentage of ED patients who smoke, EDs may play an important population-level role in motivating patients to quit smoking (3). EDs are especially important venues for reaching young and uninsured people, who appear disproportionately in EDs. The level of credibility of ED staff members in prevention and health promotion is high, and the teachable moment offered by an ED-based intervention is a persuasive argument for supporting such services (47). The core curriculum of emergency medicine now incorporates knowledge on tobacco epidemiology and motivational interviewing techniques as outlined in the joint statement of emergency medicine organizations in 2006 (3). Acknowledgment of the role of EDs in promoting preventive and health-promoting services is changing, and ED staff members readily accept that ED encounters may provide a teachable moment for encouraging smoking cessation (22,23). More knowledge is needed on how to incorporate ETC into clinical routine efficiently. Implementation of a multifaceted smoking cessation intervention in an ED can be facilitated by context-specific training, a systematic approach to assessment and action, and reminder tools (21,23,48). Routine use of computerized decision-support systems helped nurses and physicians to implement tobacco cessation treatment and further referral in a pediatric ED (49). In the clinical setting, a best-practice alert that appeared when patients were coded as smokers in the electronic health record motivated physicians to order tobacco-cessation treatment medication and to refer their patients to a telephone quitline (50). Such devices and approaches may help to further integrate tobacco control services in EDs into clinical routine.
